# Doctors’ views about training and future careers expressed one year after graduation by UK-trained doctors: questionnaire surveys undertaken in 2009 and 2010

**DOI:** 10.1186/s12909-014-0270-5

**Published:** 2014-12-21

**Authors:** Jenny J Maisonneuve, Trevor W Lambert, Michael J Goldacre

**Affiliations:** UK Medical Careers Research Group, Nuffield Department of Population Health, University of Oxford, Old Road Campus, Oxford, OX3 7LF UK

**Keywords:** Medical careers, Junior doctors, Medical education, Foundation training

## Abstract

**Background:**

The UK medical graduates of 2008 and 2009 were among the first to experience a fully implemented, new, UK training programme, called the Foundation Training Programme, for junior doctors. We report doctors’ views of the first Foundation year, based on comments made as part of a questionnaire survey covering career choices, plans, and experiences.

**Methods:**

Postal and email based questionnaires about career intentions, destinations and views were sent in 2009 and 2010 to all UK medical graduates of 2008 and 2009. This paper is a qualitative study of ‘free-text’ comments made by first-year doctors when invited to comment, if they wished, on any aspect of their work, education, training, and future.

**Results:**

The response rate to the surveys was 48% (6220/12952); and 1616 doctors volunteered comments. Of these, 61% wrote about their first year of training, 35% about the working conditions they had experienced, 33% about how well their medical school had prepared them for work, 29% about their future career, 25% about support from peers and colleagues, 22% about working in medicine, and 15% about lifestyle issues. When concerns were expressed, they were commonly about the balance between service provision, administrative work, and training and education, with the latter often suffering when it conflicted with the needs of medical service provision. They also wrote that the quality of a training post often depended on the commitment of an individual senior doctor. Service support from seniors was variable and some respondents complained of a lack of team work and team ethic. Excessive hours and the lack of time for reflection and career planning before choices about the future had to be made were also mentioned. Some doctors wrote that their views were not sought by their hospital and that NHS management structures did not lend themselves to efficiency. UK graduates from non-UK homes felt insecure about their future career prospects in the UK. There were positive comments about opportunities to train flexibly.

**Conclusions:**

Although reported problems should be considered in the wider context, in which the majority held favourable overall views, many who commented had been disappointed by aspects of their first year of work. We hope that the concerns raised by our respondents will prompt trainers, locally, to determine, by interaction with junior staff, whether or not these are concerns in their own training programme.

**Electronic supplementary material:**

The online version of this article (doi:10.1186/s12909-014-0270-5) contains supplementary material, which is available to authorized users.

## Background

Modernising Medical Careers (MMC), the current training programme for junior doctors, was introduced in the United Kingdom (UK) in 2005 [1]. MMC provides medical graduates with a two-year Foundation Programme, which replaced the single house officer year. The Foundation Programme offers a fuller training in generic skills and gives trainees general experience and core competencies in medical practice before they make their final decisions about their future specialism. Details of the Foundation Programme are available on the web at http://www.foundationprogramme.nhs.uk. The Programme is described as “a two-year generic training programme which forms the bridge between medical school and specialist/general practice training”. The two years of Foundation training immediately follow graduation in medicine and are commonly referred to as the F1 and F2 years. In the F1 year in particular, trainees begin to take supervised responsibility for patients and to consolidate skills learned at medical school.

Foundation trainees have been surveyed about their training on a number of occasions [2,3], but surveys are often highly structured, formulaic and restricted in scope. If provided with the opportunity to comment in their own words, junior doctors may communicate about issues which are unanticipated by researchers using closed format survey instruments and questionnaire formats. Although such data are sometimes criticised for being opportunistic, the results may provide valuable insights into views that survey participants wish to express. We knew from previous studies of junior doctors that we might anticipate comments on quality of training, whether the doctors felt well prepared by their medical schools for starting medical work, on long hours and exhaustion [4-7]. However, we wanted to know what these recent cohorts of doctors would tell us in their spontaneous comments. Ours is a qualitative study. As such, the researchers do not seek to infer from the comments made by a minority of the respondents what the views of the majority may be. The value of the study reported here is in identifying issues about aspects of the first year of the Foundation Programme which have been raised by some doctors, nationally, that may be worth exploring locally in future, by trainers of junior doctors, to assess whether or not they are local concerns.

The researchers applied textual analysis to comments made by over 1600 UK medical graduates, from across the UK, who graduated in 2008 and 2009 and who responded towards the end of their first postgraduate year in training, the F1 year, in 2009 and 2010 respectively. The research aims were to identify the main issues which doctors raised in spontaneous comment and to report representative comments which illustrated the issues raised.

## Methods

The UK Medical Careers Research Group (UKMCRG) sent a postal questionnaire to all UK medical graduates of 2008 and 2009 towards the end of their F1 years in 2009 and 2010. Contact addresses were provided by the General Medical Council (GMC) for all graduates who had consented to the GMC to be approached by researchers. In covering letters, each doctor was given a website address and a unique username and password to enable him or her to complete the survey online if preferred. Up to four reminders were sent to non-responders. Return of the completed questionnaire was regarded as consent to participate. Doctors who replied asking not to be contacted further were marked as non-participants and were not sent further reminders.

The questionnaire contained nine closed questions (some of which were multi-part) on career choices and factors influencing career choice, career plans, and experience of the F1 year as a whole, in addition to a section requesting demographic information. The last page of the questionnaire was headed ‘Additional Comments’ and asked the responder to ‘*Please give us any comments, if you wish, on any aspect of your training or work*’. The page contained a large blank box for responders to write their comments in their own words; the equivalent online version of the survey featured a large scrolling text box to permit extended comments to be supplied. The direction given to the responders on what they might include in their comments was ‘*We are interested, for example, in any comments about (a) medical school experience, (b) foundation year experience, (c) future career choice or job prospects, (d) working in medicine*’. However, the box for replies was unstructured and was not subdivided to reflect this suggestion, or to in any way restrict respondents to these four broad themes. Some responders structured their comments under (a), (b), (c), (d) as above, while others provided running text without referring to these subheadings. The researchers provided assurances to doctors that ‘*Your individual comments will remain confidential to researchers in UKMCRG*’. Direct personal identifiers were removed from the substantive data and comments.

All handwritten comments were transcribed into our database exactly as written, and were combined with the electronic comments entered directly by web responders. The closed questions asked by us included some that were relevant to the comments made and we summarise responses to these in the Results.

All comments were read in parallel independently by two senior UKMCRG administrative staff and a list of distinct themes and associated key words and phrases was formed by each and combined into an agreed list. The comments were then re-read by the authors and the list of themes was refined further and additional keywords identified where this was felt necessary. Keyword searches of the comments were performed to establish relative frequencies of occurrence of different themes in the data, and a process of constant comparison was used to determine whether the keywords were capturing all relevant comments on a theme; keyword lists were expanded as required. Grouping of themes into subthemes was undertaken, and themes were clustered into axial or ‘root’ codes to provide a clearer structure for interpretation. Each individual comment was coded against the coding scheme, and the frequency of comments on each topic was reported as counts, as percentages of those who commented, and as percentages of all responders. Representative excerpts from the comments are reproduced, below and in Additional file 1, using the exact words of the respondents, in order to illustrate the typical features of the themes raised.

Ethical approval: The UKMCRG surveys are approved by the National Research Ethics Service, following referral to the Brighton and Mid-Sussex Research Ethics Committee in its role as a multi-centre research ethics committee (ref 04/Q1907/48).

## Results

### Response

The response rate for the 2008 cohort was 49.3% (3302/6704), and for the 2009 cohort it was 46.7% (2918/6248). Excluded from denominators, above, were 82 doctors in the 2008 cohort and 295 in the 2009 cohort who were untraceable, and 9 and 22 respectively who replied declining to participate. Women replied in greater numbers than men (2008 cohort: men 43.3% (1140/2632), women 53.1% (2162/4072); 2009 cohort men 41.6% (999/2401), women 49.9% (1919/3847)).

Some respondents (2008: 466 doctors; 2009: 371 doctors) completed a short form of the questionnaire which did not ask responders to provide written comments. Of the 2008 cohort, 2836 respondents completed the full questionnaire and 823 wrote comments about the F1 year; this represented 29% of these responders, or 12% of the whole cohort. Of the 2009 cohort, 2551 respondents completed the full questionnaire and 793 provided comments, representing 31% of respondents or 12% of the entire cohort.

### Comparison of those who commented and those who did not

The researchers investigated whether the doctors who wrote comments differed from those who did not with respect to their experiences and views of the F1 year as expressed in the closed comments (Table 1). Statistically significant differences were found between the two groups - those who made comments and those who did not - in each of four attitude statements about the F1 year (Table 1). However, in most cases the differences, though statistically significant, were small. The exceptions were that those who commented were less likely than those who did not comment to agree that *Training was of a high standard* and that *The educational opportunities were good*. Respondents were also asked *How much have you enjoyed the F1 year overall on a scale from 1 (didn’t enjoy it at all) to 10 (enjoyed it greatly)?* Respondents who provided comments had a mean score of 6.9 compared with 7.4 for those who did not comment (t = 7.6, p < 0.001). To the question *How satisfied are you with the amount of time the F1 year has left you for family, social; and recreational activities, on a scale from 1(not at all satisfied) to 10 (extremely satisfied)?* those who commented gave a mean response of 5.8 while those who did not comment gave a mean score of 6.1 (t = 5.1, p < 0.001).

**Table 1:** **Percentage distribution of responses to closed statements for doctors who did (‘yes’), and doctors who did not comment (‘no’), and numbers on which percentages are based: 2008 and 2009 graduates combined**

		**Comments provided?**				
		**Percentages**		**Numbers**		
**Statement**	**Response**	**Yes**	**No**	**Yes**	**No**	**p-value**
I was expected to perform too much routine non-medical work	Agree	55.9	51.6	889	1941	χ^2^_2_ = 9.0
	Neither	29.7	33.4	472	1257	p = 0.011
	Disagree	14.5	15.0	230	565	
	Total	100	100	1591	3763	
I had to perform clinical tasks for which I felt inadequately trained	Agree	17.7	17.9	281	671	χ^2^_2_ = 15.5
	Neither	26.6	31.7	424	1192	p < 0.001
	Disagree	55.7	50.4	886	1895	
	Total	100	100	1591	3758	
Training was of a high standard	Agree	22.9	32.2	364	1209	χ^2^_2_ = 112.0
	Neither	45.5	48.8	724	1833	p < 0.001
	Disagree	31.6	19.1	503	718	
	Total	100	100	1591	3760	
The educational opportunities were good	Agree	31.5	38.4	499	1441	χ^2^_2_ = 60.3
	Neither	34.4	37.7	545	1418	p < 0.001
	Disagree	34.0	23.9	539	898	
	Total	100	100	1583	3757	

Neither = neither agree nor disagree.

### Classification of the comments

**Figure 1:**
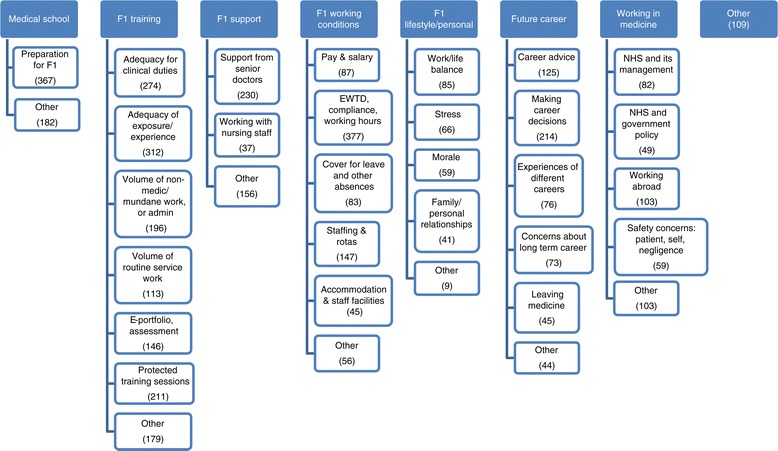
**Classification scheme used for comments and numbers of comments made under each theme.** Footnotes: EWTD denotes The European Working Time Directive. F1 denotes the Foundation Programme Year 1.

Four main themes were identified that described the comments of Foundation year doctors: aspects of *medical school*, and the preparation offered for F1; the *F1 year* itself, including training, support from peers and others, working conditions and personal/lifestyle issues; *future career*; and *working in medicine*. Further coding of these themes revealed refined subject areas (Figure 1).

### Frequency with which themes were mentioned (Figure 1, Additional file 2)

Figure 1 shows the number of comments about each theme over the two cohorts combined. Additional file 2 shows the numbers separately for the 2008 and 2009 graduates, the percentages of the respondents who commented which the numbers represent, and the percentages of all respondents (including those who did not comment) that the numbers represent.

The theme which doctors commented on most frequently was *F1 training*. Considering those who commented, 56.7% (467 doctors) and 65.1% (516 doctors) of those in the 2008 and 2009 cohorts mentioned F1 training, respectively (p < 0.001). Seven subthemes were identified within F1 training (Additional file 2, Figure 1). *Adequacy of exposure/experience* (312 comments) and *Adequacy of preparation by medical school for clinical duties* (274 comments) were the most frequent, followed by *Protected training sessions* (211 comments). The *Volume of non-medical/administrative work* was mentioned 196 times, and the *Volume of routine clinical service work* 113 times. Issues with the *e-Portfolio* system (see below) and with *assessment* more generally were the subject of 146 comments.

*F1 working conditions* was the next most frequently mentioned theme, and was referred to by 38.2% (314 doctors) and 31.9% (253 doctors) of those giving comments in the 2008 and 2009 cohorts respectively (P < 0.001). Considering both cohorts combined, the most frequently mentioned individual sub-topic was the *European Working Time Directive (EWTD), compliance with it, and working hours*, raised by 23% of those who made comments.

Considering the 2008 and 2009 cohorts combined, experience at medical school (33%, 534 doctors), future career (29%, 473 doctors), support provided to them in the F1 year (25%, 407 doctors), and working in medicine (22%, 349 doctors) were other frequently mentioned topics (Additional file 2).

Preparation by their medical school for the F1 year was mentioned by 23% of doctors who commented. Other commonly mentioned sub-topics included support from senior doctors (14%), and the need to make career decisions about future specialty too soon (13%). Smaller numbers mentioned other topics (Figure 1, Additional file 2).

Comments were not exclusively negative and we have sought to report a balance. Some comments combined ‘good’ and ‘bad’ aspects of the F1 year, from the responder’s viewpoint.

### Quotations

In Additional file 1, exemplar quotations are reproduced exactly as written, selected to illustrate the main issues and themes raised by respondents. Each quotation is given a unique numerical identifier, where the prefix “08” indicates a comment made by a 2008 graduate and “09” a comment made by a 2009 graduate, and within each cohort quotations are numbered in sequence 01,02 etc. in the order in which they appear in the Results. Text which follows includes abstracts from the quotations with these identifiers: to read the full quotation, view Additional file 1.

### Major themes

1. Medical school

Doctors reflected on the limits to preparedness for clinical practice. A 2009 graduate explained that ‘Medical school prepares you well with a good knowledge base, but it is near impossible to prepare for the job ahead - when you are put in difficult challenging clinical situations’ (09–01).

An increase in integrated ward experiences including shadowing was widely supported: ‘overall training at medical school was very good but more shadowing time would have helped’ (09–02), as was an increase in practical training (‘[medical students] should be prepared as nurses & midwives are, to do the job from day 1’. (08–01)).

Some thought that the teaching of topics such as communication skills and inter-professional skills should not be at the expense of core medical knowledge. Examples include ‘too much focus in medical education on qualities such as communication & personal qualities of a doctor & not enough focus on core basic medical knowledge’ (09–03) and ‘medical school needs to focus on providing a solid foundation of physiology and anatomy. Less focus on communication skills in the first few years is required’ (08–02).

Doctors wrote about the need to have taken Advanced Life Support (ALS) training prior to beginning F1. An illustrative comment was ‘I believe ALS training should be an obligatory component of undergraduate training… I did not get this opportunity and felt thoroughly unprepared in some situations during my first job in acute medicine’ (09–04).

Some doctors considered the quality and promptness of feedback they received for coursework in medical school to have been inadequate. A typical comment was ‘very poor levels of feedback especially with regard to written assignments’. The same doctor was ‘asked to write essays, reports throughout my medical career … for many [I] never even received a grade’ (09–05).

2) The F1 year

#### Training

Some doctors wrote that they were not given ‘enough experience in practical procedures’ (09–06) and that the high volume of routine administrative work meant that ‘I’m a glorified secretary… [and] barely get a minute to practice medicine’ (09–07).

Some doctors explained their attitudes towards the formal teaching component of the Foundation Programme. Teaching sessions were seen as a conflicting priority for trainees and, while they are compulsory sessions, doctors often commented that the subject matter was not relevant to their needs. One doctor said that ‘I was quite disappointed with the teaching programme … much time was given over to audit, ethics, law, protocols etc. … insufficient time [was] spent on learning & developing clinically …’(08–03). Training was sometimes ‘interrupted by consultant bleeps [beeps/pager alerts] which you cannot reject’ (08–04).

One doctor remarked that the NHS treated junior doctors as ‘service providers, without investing any time in our welfare or future training’ (08–05). Another felt that doctors ‘must be educated and given the opportunity to develop as doctors, not as a combined clerical and clinical support worker’ (09–08).

#### Variability of training posts

Doctors described their training experiences as variable, based on the fact that some four month training posts were better or worse than others. One doctor described this as ‘luck of the draw’ (09–09). The concept of ‘luck’ was also expressed in relation to the training experiences provided by senior doctors, which, said one doctor, ‘definitely depend on individual senior doctors, some extremely willing to teach/supervise, others are not, & it's often luck who you get’ (09–10).

#### e-Portfolio

The e-Portfolio, a personal collection of electronic evidence of career progression maintained by each junior doctor, is used as a multi-functional tool by the trainees to document progress against competencies, to reflect upon learning experiences, and to document abilities and achievements. It was common for doctors to describe their experience with the e-Portfolio system. Some doctors thought e-Portfolios were ‘a good move’ and led to ‘some of the best learning moments this year’ (09–11) , while others felt that trainees who worked in posts that were not busy ‘had the opportunity to compile extensive portfolios of reflection, assessment and other evidence, despite relatively scanty clinical contact’ (09–12).

#### Support

Junior doctors’ references to ‘support’ in the Foundation Programme varied. Some referred to support received from senior doctors, nurses, fellow junior doctors, and other administrative/managerial staff. The feeling of not being supported was often mentioned alongside staffing shortages, as in the comment from one doctor who wrote ‘Hospital too busy and understaffed… little or no support from seniors’ (08–06) and another who wrote that in one post the hospital ‘was so understaffed I often felt unsupported and concerned about patient safety’ (08–07).

Senior support could exist, however, without continuity of work: one doctor had been ‘rarely stuck without senior support when needed’ but also remarked that the ‘main downfall is lack of continuity of work with seniors’ (09–13). The lack of continuity expressed by this doctor was also reflected in comments about team building. One doctor wrote about being ‘rotated around several wards, resulting in a lack of being part of any team’ (09–14), and another at times felt ‘completely unsupported due to the lack of identifiable seniors to consult about problems’ (09–15).

Some doctors specifically mentioned nurse practitioners who ‘seem to be rapidly encroaching on the roles of FY1 doctors & in some circumstances they reduce opportunity for training/learning’ (09–16).

#### Working conditions and lifestyle issues

Trainees frequently complained that they were working beyond their contracted hours. One doctor wrote that ‘in practice simple recognition and thanks that hours can be long/longer than planned’ (08–08) would be encouraging.

A 48 hour work week was implemented as part of the European Working Time Regulations (EWTR), as the implementation of the European Working Time Directive (EWTD) in the NHS was called. EWTR provisions were often said to be ignored. All NHS Hospital Trusts in the UK are required to ensure that their employment conditions for doctors-in-training comply with the EWTR, the aim of which is to promote the health and safety of the European workforce by regulating the number and pattern of hours worked. EWTD also stipulates that workers are entitled to 11 hours of continuous rest in every 24 hour period (limiting shift length to a maximum of 13 hours). Doctors often mentioned low staffing levels in conjunction with working hours as they were cited as a reason why they work over their hours. The UKMCRG has written a more detailed study of comments on this subject elsewhere [4].

Respondents wrote that more doctors are required: one wrote ‘it is not enough to merely cut hours, there needs to be more staff’ (08–09), another ‘I have concerns regarding working time directives, as there are no extra doctors to compensate’ (08–10), and a third ‘If they employed some more doctors and let them have more control over their working lives and environments, things might improve’ (09–17). Some doctors compared their lot favourably with those of previous generations. One wrote ‘The quality of life is greatly improved as a working doctor compared to stories of older trainees. However, being a doctor is still a lifestyle and career choice.’ (09–18).

‘Flexible training’ in the NHS is training on a less than full time basis for doctors who are unable to work full time due to family commitments or for health reasons. Doctors commented positively on their experience with flexible training. One was ‘Very happy with provision of less than full time training, which has helped myself and my family considerably’ (08–11) and another described her flexible training as a ‘fantastic scheme which should be more widely available to allow young mothers like myself to progress in the medical profession’ (08–12).

The pay of doctors and the removal of free accommodation were mentioned. ‘Pay and flexibility within the NHS are the key anti-motivators. Foundation pay has been (effectively) greatly reduced by the loss of free accommodation. The NHS and my Trust seem to take every possible opportunity to decrease our pay’ (09–19).

A more general organisational issue was raised by a doctor who said ‘Foundation training should be structured in such a way as to allow for study leave, taster weeks and bleep free protected teaching … Foundation trainees are expected to have an impressive CV and be interview ready a little over a year after commencing work as a doctor. This is a ridiculously short time frame…’ (09–20).

3) Future career

Most doctors are expected to start specialty training at the beginning of the third year after medical qualification. This means applying about fifteen months after medical qualification. Some doctors felt they are required to make a decision on their choice of specialty career too early without having adequate exposure to different specialties: one wrote that ‘being force[d] into a lifelong career decision at aged 24 is far too early’ (09–21), and another felt ‘forced to pick careers before we are given a chance to experience the different options available’ (08–13). Common phrases used to describe future career decisions by trainees were phrases such as ‘forced to choose too soon’, ‘rushed to make decision’, ‘pressure to decide’. In this respect, doctors explained taster sessions and careers advice to be helpful yet not an adequate replacement for actual experience. Accordingly, some F1 doctors commented that these were reasons to want to gain work experience abroad.

4) Working in medicine and in the NHS

The management, work culture, and operation of the NHS were frequently mentioned. The underlying message of many comments was that junior doctors had to adapt and ‘work around’ the system as they found it, rather than participate in cooperative resolution of problems. One wrote ‘There is very little proper management structure within the NHS… there is a great culture to work round problems’ (09–22) and another that ‘Over my FY1 year I have not once seen a manager/matron/consultant ask a junior doctor ‘is there anything we can do to improve things?’ (09–23).

It was common for doctors who were from non-UK countries but who trained in the UK to mention government’s policy on overseas doctors. One wrote of ‘unending changes in immigration diktats from the Home Office/DoH [Department of Health] on whether I can train for a specialty in the UK on an equal footing to my peers from medical school’ (09–24) and another that ‘recent immigration changes have greatly hampered my career prospects, as I am only considered for training positions under very strict 'near-impossible' criteria for someone my age … hence my strong considerations to move abroad’ (08–14).

## Discussion

The comments received from doctors raised a number of important issues which are organised here into four key themes *Medical school, Foundation year* (including training, support, working conditions, and lifestyle/personal), *Future career,* and *Working in medicine*. While our previous research has shown that recent cohorts of junior doctors are more satisfied with their job than previous cohorts [5], our analysis of doctors’ comments reveals that there are still important concerns to be addressed. A recent commentary by Watts [6] summarises numerous concerns about the Foundation Programme and provides a perspective on its reform [6]. The need to choose a specialty too soon, loss of team structure, and the ‘e-portfolio’ assessment system are issues raised by Watts [6] and have also been expressed in the comments to us from the 2008 and 2009 cohorts.

### Preparation by medical school

In their comments, doctors considered whether medical school prepared them for Foundation training. The authors have previously published findings on the preparedness of the 2008 and 2009 cohorts one year after graduation [7]. We reported that 83% of respondents (N = 5369) stated that their medical school had prepared them well [7]. While this represents an improvement from those cohorts graduating in 1999–2005, the level of preparedness did vary by medical school attended. Overall, the lack of preparation was rated a serious problem for 2.7% and a medium-sized problem for 22.6% of respondents. In addition, women and non-white respondents were less likely to report feeling prepared for work, after adjusting for other subgroup factors [8]. The comments in the present study emphasised the value of shadowing - time spent, as a senior medical student, working with a doctor - to prepare for the F1 year, and the need to prioritise the acquisition of core medical skills and knowledge above non-technical areas such as communication and interpersonal skills. Training in life-support prior to beginning the F1 year was one specific need which some doctors mentioned in their comments. As long ago as 2001, a study surveyed 24 of 27 UK medical schools and found that 92% of schools offered some form of obligatory life-support training [9]. It was therefore surprising to find doctors telling the researchers that this was not universal almost a decade later.

### Learning and service provision by Foundation trainees

The formal role of the trainee within the Foundation Programme is as a postgraduate learner. Trainees are expected to heighten their clinical skills and demonstrate core competencies of clinical practice. Mandatory teaching sessions are also scheduled as part of the Foundation Programme. Some doctors commented that the content of these sessions did not reflect their actual needs and that attendance created conflict with service demands and priorities. Further, they felt that much of the work they did was related to the need to process routine administrative and service tasks rather than to enhance their skills and to provide learning experiences.

This echoes findings from Foundation Programme evaluation work commissioned by Medical Education England, the body with responsibility for oversight of medical education in England (now Health Education England). They found there was confusion about the role of the trainee – in their words there was “risk that [the] long-term educational mission of the service will be inappropriately dominated by short-term service requirements” [10].

In their comments doctors alluded to elements of medical training that are learnt ‘on the job’ rather than in a formal classroom setting. Previous research has qualitatively studied the workplace learning of newly qualified doctors [11,12]. Illing [11] interviewed students from three medical schools in the UK (N = 92) and found that some Foundation doctors were insufficiently prepared in skills including ward work, working on call, time management and dealing with paperwork [11]. The authors explain that these skills develop through ‘experiential learning’.

In addition, Sheehan [12] did qualitative interviews with first year junior doctors in New Zealand to better understand informal learning that takes place in the workplace [12]. The authors describe the need for junior doctors to acquire project management skills including dealing with paperwork and administration, as well as developing interpersonal skills including teamwork and liaison, management and communication. The authors point out that, though attitudes towards administrative duties are often negative, these skills are vital for the development of project management abilities, ensuring that things move forward, and for the development of prioritisation and organisation skills. Our previous study of the Foundation year doctors of 2008 and 2009 reported that many respondents felt unprepared for administrative tasks [7]. Comments in the present study concerning routine administration referred to the volume that junior doctors do, often at the expense, as the doctors saw it, of clinical work. Interestingly, similar comments were made about the volume of routine administration twenty years ago when the 1993 cohort of doctors were surveyed as pre-registration house officers [13]. For example, one doctor in 1994 commented “I spend more time on mundane administrative tasks than with patients”.

Additional training concerns reported in our analysis include the use of workplace based assessments, and reservations about the inconsistent use of electronic records of achievement. Concerns regarding ‘e-Portfolio’ have been noted elsewhere, as follows [2,14,15]. A survey and focus groups of second year Foundation doctors in Northern Ireland in 2009 found that the reason for negative views about the assessments were that assessments were often rushed, trainers and trainees did not know about the aims and requirements of them, and that the results of assessments were biased by friendship and social interactions with assessors [14]. Alarmingly, in a separate survey, 90% of junior doctors surveyed also replied that ‘incompetent trainees could obtain satisfactory results from the FY workplace based assessments’ [2].

### Working in medicine

In our study there were reservations about the management of working hours, mostly but not exclusively linked to the implementation of European Working Time Regulations. Some respondents wanted better arrangements for study leave, and the provision of opportunities to experience different specialisms as they considered their future career choices. It was felt difficult by some to reconcile the conflicting demands of the F1 year alongside having time for careful consideration of their future career. Some doctors expressed a feeling of having little influence on the system and a level of discontent with arrangements for feedback to seniors. Gilbert et al. [16] studied the views of junior doctors in an online survey of 1479 doctors (39.3% response rate). They found that doctors did not feel that their organisation was receptive to their views. For example, while 91.2% of respondents had ideas to improve their workplace, only 10.7% had their ideas implemented. When asked ‘how much do you feel valued by the NHS’, 79.3% answered that they felt either ‘not valued at all’ or ‘sometimes valued’ [16]. Dean [2] found that under half of respondents (n = 1042) felt that the Foundation Year regulatory process did not give them an adequate opportunity to highlight training problems, with approximately a third who were unsure or held neutral views [2].

### Strengths and limitations

This is a national survey of the 2008 and 2009 cohorts of medical graduates from all UK universities in 2009 and 2010. We surveyed doctors about their education, training, and career choices. We also provided doctors with the opportunity to tell us, in their own words, about any relevant issues related to their career in medicine. The ‘free-text’ nature of the comments brought to light topics of interest which we could not anticipate. Of course, many respondents chose not to comment and consequently the comments made are not necessarily representative of the views of the entire cohort. The value of the comments is to gain insights into, and consider examples of, what doctors want to express concerns about. The balance between training, administration and service provision was a frequent topic of concern, with many doctors reporting excessive demands on their time from routine administrative work.

## Conclusions

Although issues raised by the doctors should be seen in the wider context, in which the majority held favourable overall views, many who commented wanted to express disappointment in some aspects of the year. We hope that the concerns raised by our respondents will prompt trainers and those who supervise junior doctors, locally, to determine, by interaction with junior staff, whether or not these are concerns in their own training programme.

## Additional file

Additional file 1:
**Quotations referred to in the Results.**
Additional file 2:
**Numbers and percentages of comments made under each theme and subtheme, 2008 and 2009 cohort.**
