# Supplementary material for: Doctors’ views about training and future careers expressed one year after graduation by UK-trained doctors: questionnaire surveys undertaken in 2009 and 2010
Source: BMC Med Educ. 2014 Dec 21;14:270. doi: 10.1186/s12909-014-0270-5 (PMC4302441; doi:10.1186/s12909-014-0270-5)
Supplement: Additional file 1: — Quotations referred to in the Results. [file 12909_2014_270_MOESM1_ESM.docx]

**Additional file 1. Quotations referred to in the Results**

**Medical school**

| Ref. | Quotation |
| --- | --- |
| 09-01 | Medical school prepares you well with a good knowledge base, but it is near impossible to prepare for the job ahead - when you are put in difficult challenging clinical situations. |
| 09-02 | Overall training at Medical School was very good but more shadowing time would have helped. |
| 08-01 | They [medical students] should be prepared as nurses & midwives are, to do the job i.e. (FY1) from Day 1. Not start leaving the practicalities of it once they start F1. |
| 09-03 | I think there is too much focus in medical education on qualities such as communication & personal qualities of a doctor & not enough focus on core basic medical knowledge. |
| 08-02 | Medical school needs to focus on providing a solid foundation of physiology and anatomy. Less focus on communication skills in the first few years is required as this should be more of a focus in clinical years. |
| 09-04 | I believe ALS [Advanced Life Support] training should be an obligatory component of undergraduate training, after finals have been completed, immediately prior to starting F1 jobs. I did not get this opportunity and felt thoroughly unprepared in some situations during my first job in acute medicine…. |
| 09-05 | Medical School - very poor levels of feedback especially with regard to written assignments. I do not see the point of being asked to write essays, reports throughout my medical career that I have never received feedback from to improve and for many never even received a grade. |

**F1 training**

| Ref. | Quotation |
| --- | --- |
| 09-06 | Both medical school and FY1 year do not give enough experience in practical procedures. Much of F1 work involved far too much admin work with little to no educational benefit and not enough real clinical experience… |
| 09-07 | I`m an F1 - aka a glorified secretary. I spend my life doing the work a discharge co-ordinator should do, filling in paperwork and other pen pushing tasks. I barely get a minute to practice medicine… |
| 08-03 | I was quite disappointed with the teaching programme & although time was allocated for teaching, the structure & contents were not as helpful as hoped in developing our clinical knowledge & skills, to developing as doctors. Much time was given over to audit, ethics, law, protocols etc. which although important I felt too much time & emphasis were placed on those with insufficient time spent on learning & developing clinically preparing for postgraduate exams & teaching to increase our knowledge & understanding of their application would have been much more valuable. |
| 08-04 | Minimal teaching/educational opportunity, with whatever 'bleep free' time we had (1hr/week) interrupted by consultant bleeps [beeps / pager alerts] which you cannot reject. I think seniors need to be aware that we are in a training post & entitled to learning. |
| 08-05 | The Foundation Training Programme is a joke. There is no training. The NHS certainly get their daily pound of flesh from us so called 'service providers' without investing any time in our welfare or future training. No wonder so many junior doctors leave the NHS or opt for GP training to escape. |
| 09-08 | Trainees must not be treated as commodities used to fill rotas. We must be educated and given the opportunity to develop as doctors, not as a combined clerical and clinical support worker. |

**F1 training - Variability of training posts**

| Ref. | Quotation |
| --- | --- |
| 09-09 | A lot of both foundation year and medical school is luck of the draw with regards to consultants and their teams and how they regard teaching, which is therefore not even across the board. |
| 09-10 | Training/education opportunities definitely depend on individual senior doctors, some extremely willing to teach/supervise, others are not, & it's often luck who you get… |

**F1 training - e-Portfolio**

| Ref. | Quotation |
| --- | --- |
| 09-11 | E portfolio is a good move, consultants who are keen to sit down and do proper case based discussion mean they have been some of the best learning moments this year. However I think its early days not yet fully part of the teaching culture … |
| 09-12 | I often needed to work for 11-12 hours a day. However, this paradoxically left me with fewer opportunities to provide evidence of the experience I was gaining. In contrast, those trainees with less busy jobs (for example, a surgical rotation with never more than 6 patients at once) had the opportunity to compile extensive portfolios of reflection, assessment and other evidence, despite relatively scanty clinical contact. |

**F1 Support**

| Ref. | Quotation |
| --- | --- |
| 08-06 | Hospital too busy and understaffed so majority of work was "donkey work” with little or no support from seniors and little to no opportunity to go in depth with patients and have a good understanding of their disease process… |
| 08-07 | In my first job (elderly care) the team was so understaffed I often felt unsupported and concerned about patient safety. The next two jobs were much better supported. |
| 09-13 | Most seniors very friendly & approachable, rarely been stuck without senior support when needed it. Overall enjoyed the year very much. Main downfall is lack of continuity of work with seniors… |
| 09-14 | …within the 4 month blocks I have rotated around several wards, resulting in a lack of being part of any team and a lack of sufficient educational opportunities since each week I can work with a different registrar/consultant. … |
| 09-15 | On some placements, the lack of a `team` was a problem. There was times when I felt completely unsupported due to the lack of identifiable seniors to consult about problems. |
| 09-16 | Nurse practitioners seem to be rapidly encroaching on the roles of FY1 doctors & in some circumstances they reduce opportunity for training/learning. |

**F1 Working conditions and lifestyle**

| Ref. | Quotation |
| --- | --- |
| 08-08 | I feel very strongly that the current climate of management (senior clinicians) officially stating that juniors must not work more than 48 hours, when this is impossible to achieve, and then refusing to accept evidence that this is occurring has led to a cultural change in which juniors feel obliged to protest about their working hours. In practice simple recognition and thanks that hours can be long/longer than planned might prevent this situation. |
| 08-09 | Medicine does not fit the confines of a 9-5 job, therefore in order to make doctors more effective at work it is not enough to merely cut hours, there needs to be more staff. |
| 08-10 | I have concerns regarding working time directives, as there are no extra doctors to compensate - on paper my hours are reduced. However, I cannot leave my work at 5pm. |
| 09-17 | Managers put unnecessary pressure on us to work within our 48 hours/week limits, yet this is often not possible due to the workload and lack of doctors. If they employed some more doctors and let them have more control over their working lives and environments, things might improve. |
| 09-18 | The quality of life is greatly improved as a working doctor compared to stories of older trainees. However, being a doctor is still a lifestyle and career choice. The two unfortunately go hand in hand. As I often say to friends, we don`t run a shoe shop that we can just shut at 5pm. |
| 08-11 | Very happy with provision of less than full time training, which has helped myself and my family considerably. Would be good if LTFT [*less than full-time working*] was available at more than 50%, i.e. 60-75%, as this would have given more experience earlier and enabled completion of F1 and F2 in 3 years rather than 4. |
| 08-12 | Have had a flexible training post so only half way through FY1. Fantastic scheme which should be more widely available to allow young mothers like myself to progress in the medical profession. |
| 09-19 | Pay and flexibility within the NHS are the key anti-motivators. Foundation pay has been (effectively) greatly reduced by the loss of free accommodation. The NHS and my Trust seem to take every possible opportunity to decrease our pay (for example, decreasing the banding on posts that have exactly the same hours). |
| 09-20 | Foundation training should be structured in such a way as to allow for study leave, taster weeks and bleep free protected teaching. This is not the case in the Trust in which I work. Foundation trainees are expected to have an impressive CV and be interview ready a little over a year after commencing work as a doctor. This is a ridiculously short time frame in which to have completed audits, gone on courses, explored different specialties and sat exams. Whilst it will be argued that the aforementioned are not requirements for ST applications, it is a well-known fact that those that have such points on their CV are more likely to get interviewed and subsequently offered positions. Trainees must not be treated as commodities used to fill rotas. We must be educated and given the opportunity to develop as doctors, not as a combined clerical and clinical support worker. |

**Future career**

| Ref. | Quotation |
| --- | --- |
| 09-21 | Being force[d] into a lifelong career decision at aged 24 is far too early. Bring back the old system, allow us to experience many different specialties as an SHO before deciding on long term career options, rather than after only 16 months as a qualified doctor) of which only 4 months is an SHO). |
| 08-13 | I feel like we are being forced to pick careers before we are given a chance to experience the different options available. |

**Working in medicine and in the NHS**

| Ref. | Quotation |
| --- | --- |
| 09-22 | There is very little proper management structure within the NHS, the most fundamental missing part being the assessment and match of job workload to resource. … The most frustrating thing I find in the NHS is that there is a great culture to work round problems, rather than actually investing a little time to finally deal with them! |
| 09-23 | There needs to be a shift in culture in how doctors feedback on how to improve operational and clinical issues to improve patient care. Junior doctors moan about a lot of issues but don`t know who to approach or cannot be bothered to make the effort. Equally management needs to be more receptive. Over my FY1 year I have not once seen a manager/matron/consultant ask a junior doctor `is there anything we can do to improve things`. I found this bizarre as we are the ones on the shop floor the most. A recent survey in our trust showed that 75% of junior doctors did not know who to approach with ideas. |
| 09-24 | Like many of my colleagues I was affected by MMC. However, I was doubly affected by continual changes in the Government`s position on foreign doctors. I trained in a London medical school. I speak perfect English. I paid overseas fees and have endured separation from my family. Yet I have had to endure unending changes in immigration diktats from the Home Office/DoH [Department of Health] on whether I can train for a specialty in the UK on an equal footing to my peers from medical school. The goalposts are constantly shifting and a lack of clarity on this issue has caused much anxiety. The quota-limited 7% of UK medical students from non-EU countries should be allowed to complete specialty training without having to jump through endless loopholes and being made to feel like 3rd class illegal aliens. This, more than anything else, would make me despair over a job I have devoted myself to. |
| 08-14 | As foreign UK medical graduate, I was always re-assured of a training job. Now recent immigration changes have greatly hampered my career prospects, as I am only considered for training positions under very strict 'near-impossible' criteria for someone my age (e.g. must have a Master's degree, etc). Very disappointing, hence my strong considerations to move abroad. |
