# Supplementary material for: Doctors’ views about training and future careers expressed one year after graduation by UK-trained doctors: questionnaire surveys undertaken in 2009 and 2010
Source: BMC Med Educ. 2014 Dec 21;14:270. doi: 10.1186/s12909-014-0270-5 (PMC4302441; doi:10.1186/s12909-014-0270-5)
Supplement: Additional file 2: — Numbers and percentages of comments made under each theme and subtheme, 2008 and 2009 cohort. [file 12909_2014_270_MOESM2_ESM.docx]

**Additional file 2. Numbers and percentages of comments made under each theme and subtheme, 2008 and 2009 cohort**

|  |  |  |  | **2008** | | |  | **2009** | | |  | **Total** | | |
| --- | --- | --- | --- | --- | --- | --- | --- | --- | --- | --- | --- | --- | --- | --- |
| **Theme** |  | **Subtheme** |  | **n** | **Percentage commenting** | **Percentage respondents** |  | **n** | **Percentage commenting** | **Percentage respondents** |  | **n** | **Percentage commenting** | **Percentage respondents** |
|  |  |  |  |  |  |  |  |  |  |  |  |  |  |  |
| **Medical School** | | Preparation for F1 |  | 182 | 22.1 | 6.4 |  | 185 | 23.3 | 7.3 |  | 367 | 22.7 | 6.8 |
|  |  | Other |  | 80 | 9.7 | 2.8 |  | 102 | 12.9 | 4.0 |  | 182 | 11.3 | 3.4 |
|  |  | Any of the above |  | 262 | 31.8 | 9.2 |  | 272 | 34.3 | 10.7 |  | 534 | 33.0 | 9.9 |
|  |  |  |  |  |  |  |  |  |  |  |  |  |  |  |
| **F1 training** |  | Adequacy for clinical duties |  | 133 | 16.2 | 4.7 |  | 141 | 17.8 | 5.5 |  | 274 | 17.0 | 5.1 |
|  |  | Adequacy of exposure/experience |  | 137 | 16.6 | 4.8 |  | 175 | 22.1 | 6.9 |  | 312 | 19.3 | 5.8 |
|  |  | Volume of non-medic/ mundane work or admin | | 94 | 11.4 | 3.3 |  | 102 | 12.8 | 4.0 |  | 196 | 12.1 | 3.6 |
|  |  | Volume of routine service work |  | 52 | 6.3 | 1.8 |  | 61 | 7.7 | 2.4 |  | 113 | 7.0 | 2.1 |
|  |  | E-portfolio, assessment |  | 64 | 7.8 | 2.3 |  | 82 | 10.3 | 3.2 |  | 146 | 9.0 | 2.7 |
|  |  | Protected training sessions |  | 105 | 12.8 | 3.7 |  | 106 | 13.4 | 4.2 |  | 211 | 13.1 | 3.9 |
|  |  | Other |  | 93 | 11.3 | 3.3 |  | 86 | 10.8 | 3.4 |  | 179 | 11.1 | 3.3 |
|  |  | Any of the above |  | 467 | 56.7 | 16.5 |  | 516 | 65.1 | 20.2 |  | 983 | 60.8 | 18.2 |
|  |  |  |  |  |  |  |  |  |  |  |  |  |  |  |
| **F1 support** |  | From senior doctors |  | 119 | 14.4 | 4.3 |  | 111 | 14.0 | 4.4 |  | 230 | 14.2 | 4.3 |
|  |  | Working with nursing staff |  | 24 | 2.9 | 0.8 |  | 13 | 1.6 | 0.5 |  | 37 | 2.3 | 0.7 |
|  |  | Other |  | 66 | 8.0 | 2.5 |  | 90 | 11.3 | 3.5 |  | 156 | 9.7 | 2.9 |
|  |  | Any of the above |  | 203 | 24.7 | 7.2 |  | 204 | 25.7 | 8.0 |  | 407 | 25.2 | 7.6 |
|  |  |  |  |  |  |  |  |  |  |  |  |  |  |  |
| **F1 working conditions** | | Pay & salary |  | 48 | 5.8 | 1.7 |  | 39 | 4.9 | 1.5 |  | 87 | 5.4 | 1.6 |
|  |  | EWTD, compliance, working hours |  | 217 | 26.4 | 7.7 |  | 160 | 20.2 | 6.3 |  | 377 | 23.3 | 7.0 |
|  |  | Cover for leave and other absences |  | 52 | 6.3 | 1.8 |  | 31 | 3.9 | 1.2 |  | 83 | 5.1 | 1.5 |
|  |  | Staffing & rotas |  | 80 | 9.7 | 2.8 |  | 67 | 8.4 | 2.6 |  | 147 | 9.1 | 2.7 |
|  |  | Accommodation, staff facilities |  | 28 | 3.4 | 1.0 |  | 17 | 2.1 | 0.7 |  | 45 | 2.8 | 0.8 |
|  |  | Other |  | 20 | 2.4 | 0.7 |  | 36 | 4.5 | 1.4 |  | 56 | 3.5 | 1.0 |
|  |  | Any of the above |  | 314 | 38.2 | 11.1 |  | 253 | 31.9 | 9.9 |  | 567 | 35.1 | 10.5 |
|  |  |  |  |  |  |  |  |  |  |  |  |  |  |  |
| **F1 lifestyle/ personal** | | Work/life balance |  | 52 | 6.3 | 1.8 |  | 33 | 4.2 | 1.3 |  | 85 | 5.3 | 1.6 |
|  |  | Stress |  | 25 | 3.0 | 0.9 |  | 31 | 3.9 | 1.2 |  | 66 | 4.1 | 1.2 |
|  |  | Morale |  | 37 | 4.5 | 1.3 |  | 22 | 2.8 | 0.9 |  | 59 | 3.7 | 1.1 |
|  |  | Family/personal relationships |  | 26 | 3.2 | 0.9 |  | 15 | 1.9 | 0.6 |  | 41 | 2.5 | 0.8 |
|  |  | Other |  | 2 | 0.2 | 0.2 |  | 7 | 0.9 | 0.3 |  | 9 | 0.6 | 0.2 |
|  |  | Any of the above |  | 137 | 16.6 | 4.8 |  | 100 | 12.6 | 3.9 |  | 237 | 14.7 | 4.4 |
|  |  |  |  |  |  |  |  |  |  |  |  |  |  |  |
| **Future career** | | Career advice |  | 42 | 5.1 | 1.5 |  | 83 | 10.5 | 3.3 |  | 125 | 7.7 | 2.3 |
|  |  | Making career decisions |  | 85 | 10.3 | 3.0 |  | 129 | 16.3 | 5.1 |  | 214 | 13.2 | 4.0 |
|  |  | Experiences of different careers | | 49 | 6.0 | 1.7 |  | 27 | 3.4 | 1.1 |  | 76 | 4.7 | 1.4 |
|  |  | Concerns about long term career |  | 45 | 5.5 | 1.6 |  | 28 | 3.5 | 1.1 |  | 73 | 4.5 | 1.4 |
|  |  | Leaving medicine |  | 24 | 2.9 | 0.8 |  | 21 | 2.6 | 0.8 |  | 45 | 2.8 | 0.8 |
|  |  | Other |  | 23 | 2.8 | 0.8 |  | 21 | 2.6 | 0.8 |  | 44 | 2.7 | 0.8 |
|  |  | Any of the above |  | 225 | 27.3 | 7.9 |  | 248 | 31.3 | 9.7 |  | 473 | 29.3 | 8.8 |
|  |  |  |  |  |  |  |  |  |  |  |  |  |  |  |
| **Working in medicine** | | NHS and its management |  | 51 | 6 | 2 |  | 31 | 3.9 | 1.2 |  | 82 | 5.1 | 1.5 |
|  |  | NHS and government policy |  | 35 | 4.3 | 1.2 |  | 14 | 1.8 | 0.5 |  | 49 | 3.0 | 0.9 |
|  |  | Working abroad |  | 59 | 7.2 | 2.1 |  | 44 | 5.5 | 1.7 |  | 103 | 6.4 | 1.9 |
|  |  | Safety concerns: patient, self, negligence | | 33 | 4.0 | 1.2 |  | 26 | 3.3 | 1.0 |  | 59 | 3.6 | 1.1 |
|  |  | Other |  | 39 | 4.7 | 1.4 |  | 64 | 8.1 | 2.5 |  | 103 | 6.4 | 2.0 |
|  |  | Any of the above |  | 191 | 23.2 | 6.7 |  | 158 | 19.9 | 6.2 |  | 349 | 21.6 | 6.5 |
|  |  |  |  |  |  |  |  |  |  |  |  |  |  |  |
| **Other** |  | Everything else |  | 61 | 7.4 | 2.2 |  | 48 | 6.1 | 1.9 |  | 109 | 6.7 | 2.0 |
|  |  |  |  |  |  |  |  |  |  |  |  |  |  |  |
|  |  | **Total respondents** |  |  | **823**^1^ | **2836**^2^ |  |  | **793**^1^ | **2551**^2^ |  |  | **1616**^1^ | **5387**^2^ |

^1^ Number of doctors who wrote free text comments

^2^ Number of responders completing full questionnaire
